# Involvement of Small RNAs in Phosphorus and Sulfur Sensing, Signaling and Stress: Current Update

**DOI:** 10.3389/fpls.2017.00285

**Published:** 2017-03-10

**Authors:** Smita Kumar, Saurabh Verma, Prabodh K. Trivedi

**Affiliations:** ^1^Council of Scientific and Industrial Research – National Botanical Research InstituteLucknow, India; ^2^Centre of Bio-Medical ResearchSanjay Gandhi Post-Graduate Institute of Medical Sciences Lucknow, India; ^3^Department of Biotechnology, Babasaheb Bhimrao Ambedkar UniversityLucknow, India

**Keywords:** abiotic stress, gene regulation, miRNA, nutrient deficiency, nutrient homeostasis

## Abstract

Plants require several essential mineral nutrients for their growth and development. These nutrients are required to maintain physiological processes and structural integrity in plants. The root architecture has evolved to absorb nutrients from soil and transport them to other parts of the plant. Nutrient deficiency affects several physiological and biological processes in plants and leads to reduction in crop productivity and yield. To compensate this adversity, plants have developed adaptive mechanisms to enhance the acquisition, conservation, and mobilization of these nutrients under deficient or adverse conditions. In addition, plants have evolved an intricate nexus of complex signaling cascades, which help in nutrient sensing and uptake as well as to maintain nutrient homeostasis. In recent years, small non-coding RNAs such as micro RNAs (miRNAs) and endogenous small interfering RNAs have emerged as important component in regulating plant stress responses. A set of these small RNAs (sRNAs) have been implicated in regulating various processes involved in nutrient uptake, assimilation, and deficiency. In response to phosphorus (P) and sulphur (S) deficiencies, role of sRNAs, miR395 and miR399, have been identified to be instrumental; however, many more miRNAs might be involved in regulating the plant response to these nutrient stresses. These sRNAs modulate expression of target genes in response to P and S deficiencies and regulate their uptake and utilization for proper growth and development of the plant. This review summarizes the current understanding of uptake, sensing, and signaling of P and S and highlights the regulatory role of sRNAs in adaptive responses to these nutrient stresses in plants.

## Introduction

Plants acquire mineral nutrients from the soil through extensive root system for their growth and development (Lynch, 2011; Gruber et al., 2013). Conventionally, there are 16 essential mineral nutrients, which are crucial for plant development. These are required in different amounts and thus are categorized as primary, secondary, and micronutrients. Primary or the macronutrients include nitrogen (N), phosphorus (P), and potassium (K); essentially required in various processes including photosynthesis, cell division, protein synthesis, and disease resistance. Secondary nutrients comprising calcium (Ca), magnesium (Mg), and sulfur (S) are required in lesser amount than the primary nutrients by the plants. Similarly, micronutrients required in trace amounts comprise boron (Bo), chlorine (Cl), iron (Fe), manganese (Mg), molybdenum (Mo), and zinc (Zn; Schachtman and Shin, 2007). The requirement of micronutrients is as essential as the primary and secondary nutrients for plant growth and development. These nutrients are taken up by the root system from the soil and associated microorganisms such as *rhizobium* and mycorrhizal associations. Most of the absorption of nutrients from soil is performed by the root hairs that form the extreme most component of the root system. Roots being the first site to sense nutrient availability maximizes nutrient uptake using high surface area and volume ratio (Gruber et al., 2013). Thus, the plants restructure the root architecture according to the nutrient availability, e.g., the succulents have deep penetrating with developed primary root whereas the aquatic plants have sparsely distributed and less developed primary roots. Plants possess counteracting mechanisms against nutrient deficiency that includes sensing, signaling, and acquisition of nutrients and restructuring the root architecture depending upon nutrient availability in the soil. The restructuring happens in the nutrient pockets of the soils where the root hairs and secondary roots develop well to enhance nutrient absorption whereas the number and length of root hairs and other root system components decreases in the nutrient deficient regions of the soil. From the rhizosphere and root epidermal cells, nutrients are transported to the vascular cells and are further allocated to different tissues (Nath and Tuteja, 2016). Different transporters including ion channels, electrochemical potential-driven transporters, group translocators, electron carriers, and voltage gated channels present on the plasma membrane take part in the nutrient uptake and allocation in various cellular organelles and tissues (Ludewig and Frommer, 2002; Saier et al., 2016; Sasaki et al., 2016). Under nutrient deficiency, a complex signaling cascade from aerial plant tissue activates various biochemical components required for uptake and transport of nutrients to meet optimum requirement for growth and development.

In recent past, role of non-coding RNAs (ncRNAs) have been implicated in stress response including nutrient deficiency (Sunkar et al., 2007; Mao et al., 2009; Kuo and Chiou, 2011; Sharma et al., 2015). MicroRNAs (miRNAs) and endogenous small interfering RNAs (siRNAs) of 21–24 nucleotides length are two major classes of small regulatory RNAs in plants. Though these small RNAs (sRNAs) differ in their mode of biogenesis, regulate several processes through transcriptional (TGS) and post-transcriptional gene silencing (PTGS; Napoli et al., 1990; Vaucheret and Fagard, 2001; Valencia-Sanchez et al., 2006; Khraiwesh et al., 2012; Tiwari et al., 2014). A number of studies suggest that these sRNAs regulate gene expression and thus modulate plant growth and development in normal or stress conditions including nutrient deficiency (He and Hannon, 2004; Sunkar et al., 2007; Paul et al., 2015; Melnikova et al., 2016; Sharma et al., 2016; Wang et al., 2016). Plants adapt to nutrient deficient conditions by modulating the expression of genes encoding specific group of transporters and metabolic enzymes. This differential regulation of these genes is controlled by a set of miRNA families. Major research endeavors have identified several miRNAs responsive to nutrient deficiencies in different plant species including *Arabidopsis*, maize, rice, and *Medicago truncatula* (Sunkar et al., 2007). In *Arabidopsis*, miR156 family has been identified to be most responsive toward nitrogen deficiency (Liang et al., 2012). In addition, involvement of miR160 and miR170 has been shown in altering root structure architecture of plants in response to nitrogen deficiency (Liang et al., 2012). Nodule development under nitrogen deficiency has been shown to be regulated by miR169 and miR172 reported in (Combier et al., 2006; Yan et al., 2013). These miRNAs up regulate nitrate transporters under nitrogen deficiency conditions. Recent studies using genome-wide expression analysis and functional genomics approaches have identified differentially expressed miRNAs and their regulatory roles in different nutrient as well as other metal stresses including Copper (Cu), Iron (Fe), Manganese (Mn), and, Zinc (Zn). miRNAs including miR397, miR408, and miR857, have been observed to be up-regulated during Cu starvation and functions in the regulation of Cu levels in plants (Abdel-Ghany and Pilon, 2008). Under Fe-deficiency in plants, eight conserved miRNA genes of five families including miR854, were observed to be up-regulated. Analysis of *cis*-regulatory elements upstream of these miRNA genes revealed presence of IDE1/IDE2 (Iron-deficiency responsive *cis*-Elements 1 and 2) motifs in their promoter regions (Kong and Yang, 2010). Notably, the induction of miRNAs modulate expression of array of genes and thus facilitate nutrient homeostasis in plants. In this review, recent progress and updates related to sRNAs-mediated mineral nutrient uptake, sensing and signaling have been reviewed. The focus of the review is on the nutrients P and S, which are essential for the growth and stress response in plants.

## Phosphorus (P)

### Uptake and Transport System

Phosphorus, is an important constituent of many organic molecules such as nucleic acid, sugars, ATP, and phospholipids, which provides energy and help in the growth and development of living organisms. Plants acquire phosphorus in the inorganic form (Pi) by high affinity transporters (PHTs) and Pi/H^+^ symporters (Mlodzinska and Zboinska, 2016). In *Arabidopsis*, PHT gene family is divided into four groups (PHT1, PHT2, PHT3, and PHT4) are involved in different functions. PHT1;1 and PHT1;4 are high affinity transporters which acquire P from the soil, PHT1;5 is responsible for the source to sink translocation, PHT1;8 and PHT1;9 carry out root-shoot Pi mobilization (Nussaume et al., 2011; Ye et al., 2015). PHT2;1 is the low affinity transporter and is involved in the Pi translocation between root and shoot. Other Pi transporter, PHO1 (Phosphate 1), contains SPX domain and EXS [Early Responsive to Dehydration1 (ERD1)], is involved in Pi loading into the xylem as *pho1* mutant exhibits lower Pi levels in shoots (Liu et al., 2014; Wege et al., 2016). PHO2 (PHOSPHATE2; ubiquitin-conjugating enzyme E2) acts as a negative regulator of Pi uptake and degrades phosphate transporters (PHT1;1, PHT1;2, PHT1;3, PHT1;4, PHO1) and phosphate transporter facilitator1 (PHF1; Huang et al., 2013). The identification and detailed investigation of other transporters involved in Pi homeostasis is required to understand the transport, cellular metabolism, and nutrient fluctuations in plants.

The Phosphate Starvation Response 1 (PHR1) is a central regulator of Pi homeostasis and up-regulates the expression of PHT and Pi-starvation induced genes through binding to the PHR1-binding sequences in the promoter region of many Pi-related genes (Chiou and Lin, 2011; Sobkowiak et al., 2012; Wang et al., 2013). Under phosphate deficient conditions, systemic and local signaling pathways are activated for the regulation of Pi uptake, assimilation, and redistribution inside the plant (Scheible and Rojas-Triana, 2015). A series of signaling events regulated by different factors take place leading to modulation in root structure architecture/morphology by increasing root hair length as well as lateral root formation to enhance Pi uptake from the external environment (**Table 1**) (Linkohr et al., 2002; Svistoonoff et al., 2007; Fang et al., 2009; Shukla et al., 2015). In addition to above signaling events, secondary messengers such as Ca^2+^, inositol polyphosphates (IPs), and reactive oxygen species (ROS) play an important role in regulating Pi homeostasis in plants (Chiou and Lin, 2011). As root development and architecture is a complex trait, detailed studies related to hormonal cross-talk and secondary messengers will help in better understanding of the mechanism and overall plant’s efficiency to adapt and combat nutrient deficiency.

**Table 1:** Various signaling pathway components involved in Pi-related response.

Factor	Signal	Experiment	Effect	Reference
miR399 overexpression	Long distance	Vascular grafting	Suppression of PHO2; increased Pi transporter; increased Pi acquisition	Lin et al., 2008; Pant et al., 2008, 2009
Pi-deficiency in one of the root partner	Systemic/Local	Split-root	Entire sets of PSI transcripts regulated systemically; other groups of PSI gene transcripts are regulated locally	Thibaud et al., 2010
Shoot Pi concentration	Systemic	Split-root	Cluster root growth; citrate exudation (White Lupin); repression of the plant genes involved in AM symbiosis	Shane et al., 2003; Shane and Lambers, 2006; Breuillin et al., 2010; Balzergue et al., 2011
Auxin	Systemic	Exogenous application in P-sufficient roots	Mimics Pi-deficiency, i.e., reduced primary root length, higher lateral root density, root hair elongation	Gilbert et al., 2000; Williamson et al., 2001; López-Bucio et al., 2002
Ethylene	Systemic	Transcriptome analysis of Pi-deficient plants	Upregulation of ethylene responsive genes; antagonistic to auxin signaling	López-Bucio et al., 2002; Ma et al., 2003; Uhde-Stone et al., 2003; Zhang et al., 2003; He et al., 2005; Morcuende et al., 2007; Kim et al., 2008; Thibaud et al., 2010
Cytokinin	Systemic	Phosphate deficient	Repress induction of PSI genes; increase in intracellular Pi concentration	Martín et al., 2000; Franco-Zorrilla et al., 2005

### Regulation of Pi Homeostasis by Small RNA

Various studies identified an array of genes involved in the Pi signaling network mechanisms (Liang et al., 2014). In addition, involvement of sRNAs in regulating the expression of genes involved in phosphate uptake and assimilation in different plant species including *Arabidopsis*, maize, soybean, rice, and tomato has been demonstrated (Lundmark et al., 2010; Pei et al., 2013; Xu et al., 2013; Zhao et al., 2013; Gu et al., 2014). Identification of these phosphate starvation responsive miRNA families (**Table 2**) has been identified via various approaches including high throughput sRNA sequencing. These miRNAs mediate regulation of phosphate uptake, transport, and assimilation in plants through targeting a set of genes. Studies suggest that miRNA families such as miR156, miR159, miR166, miR319, miR395, miR398, miR399, miR447, and miR827 are commonly responsive to Pi-deficiency among different species and are presumably involved in conserved Pi-deficiency signaling networks (Sun et al., 2012; Sunkar et al., 2012). In most of these studies, enhanced levels of miR156, miR399, miR778, miR827, miR2111 and repressed levels of miR169, miR395, and miR398 were observed under Pi-deficiency (Hsieh et al., 2009). However, abundance of miR778 and miR2111 is reduced by approximately twofold after the addition of Pi (Pant et al., 2009). Apart from Pi-deficiency, role of miR2111 has been demonstrated in N-starvation (Liang et al., 2012). Similarly, miR827 and miR399 are responsive to N-starvation and target Nitrogen Limitation Adaptation (NLA) gene and enhances the expression of PHO2 transporter. Analysis of proximal promoters of Pi-responsive MIRNA genes suggest presence of conserved motifs which might be involved in regulated expression under Pi-deficiency (Zeng et al., 2010). Detailed characterization of these *cis*-elements and interacting proteins will help in the better understanding of the molecular regulation mechanism of genes involved in Pi acquisition and transport.

**Table 2:** Plant micro RNA (miRNA) families responsive to Pi-deficiency.

miRNA families		Plant species	Reference
Up-regulated	Down-regulated		
miR156, miR157, miR159, **miR163**, miR164, miR165, miR166, miR167, miR171, miR172, miR319, miR391, miR393, miR399, miR408, miR447, **miR778**, miR780, miR822, miR824, miR827, miR828, miR843, miR865, **miR866**	miR169, miR395, miR398, miR402, miR779, miR823, miR860, miR2111	*Arabidopsis thaliana*	Hsieh et al., 2009; Pant et al., 2009; Lundmark et al., 2010
miR399, miR827		*Oryza sativa*	Zhou et al., 2008; Lin et al., 2010
miR156, miR160, miR166, miR168, miR171, miR395, miR396, miR399, **miR437**, miR447, miR472, miR477, miR809, miR818, **miR830**, miR845, miR854, miR857, miR863, miR866, **miR896**, mir903, miR904, **miR1222**	miR159, miR164, miR166, miR167, miR319, miR390, miR395, miR396, miR397, **miR447**, miR530, miR810, miR818, miR857, miR893, miR895, **miR1211**	*Lupinus albus*	Zhu et al., 2010
miR156, miR157, miR159, miR167, miR168, miR319, miR396, miR474, miR482, miR894, miR1509	miR160, miR165, miR166, miR168, miR396, miR398, miR834, miR854, miR1118, miR1311, miR1427, miR1436, miR1450, **miR1507**, miR1508, miR1511, miR1846, miR1858, miR1879, miR1881	*Glycine max*	Zeng et al., 2010
miR156, miR157, miR170, miR319, miR393, miR399	miR160, miR167, miR169, miR317, miR397, miR398, miR408, miR1511, miR1513, miR1515, miR1516, miR2118	*Phaseolus vulgaris*	Valdés-López et al., 2010
miR171, miR172, miR394, miR395, miR398, miR399, miR779, **miR837**, miR839, miR840, miR847, miR860, miR861, miR862, miR867	miR158, miR169, miR172, miR319, miR398, **miR771, miR775**, miR158, miR169, miR319, miR172, miR771, miR775	*Solanum lycopersicum*	Gu et al., 2010

*Unique miRNAs are indicated in **Bold**.*

Genes encoding SPX subfamily proteins, SPX-MFS (Major Facility Superfamily) are reported to be involved in the Pi-sensing/transport and are targeted by miR827 (Pacak et al., 2016). In addition, miR398a has been shown to regulate the expression of a set of genes under P, N, and C deficiency and helps in the maintenance of mineral balance in plants (Dugas and Bartel, 2008). Studies also suggest that phloem enriched sRNAs respond to P-deficiency. Grafting studies carried out on *Arabidopsis* wild type and the miRNA processing *hen1-1* mutant plants identified miR399 and miR395 as phloem sap sRNAs that are transported from shoot to root and target genes in the roots of the seedlings exposed to nutrient deficiency (Buhtz et al., 2010). miRNA-mediated post-transcriptional and ubiquitin-mediated post-translational regulatory pathways have been shown to modulate Pi transport activity in response to external Pi status (Lin et al., 2014). Interestingly, responsiveness of many miRNAs is species- and tissues/organs-specific under Pi-deficiency. In response to phosphate starvation, miR395 is down-regulated in the *Arabidopsis* shoots but up-regulated in the shoots of white lupin (*Lupinus albus*; Zhu et al., 2010).

Among all Pi-responsive miRNAs, miR399 is the most studied phosphate starvation responsive sRNA which is up-regulated under Pi-stressed conditions (Bari et al., 2006; Chiou et al., 2006; Phillips et al., 2007). In *Arabidopsis*, all the six members of MIR399 genes (MIR399A–F) are induced under Pi-deficiency (Kuo and Chiou, 2011). Overexpression of miR399 in transgenic *Arabidopsis* led to the enhanced Pi uptake and allocation to the shoots (Aung et al., 2006; Chiou et al., 2006). Interestingly, overexpression of *Arabidopsis* miR399 in tomato exhibited increased Pi accumulation and secretion of acid phosphatase in the roots causing hydrolysis of soil organic P and dissolution of Pi (Gao et al., 2010). Studies suggest that miR399 targets three genes; Pi transporter (PHT1;7), a DEAD box helicase and PHO2 which encodes putative ubiquitin-conjugating enzyme (UBC) under Pi deficient condition (Fujii et al., 2005). miR399 acts as a positive regulator and enhances Pi uptake and translocation under Pi deficient condition, while PHO2 functions as a negative regulator and suppress these activities to prevent excess Pi uptake under Pi sufficient condition (Lin et al., 2008; Yuan and Liu, 2008). In addition, miR399 also serves as a long-distance signal from shoot to suppress PHO2 expression and maintain Pi homeostasis in plants. Furthermore, miR399 has been shown to function in multiple nutrient deficiency responses in rice. GeneChip analysis of the OsmiR399 overexpressing plants revealed up regulation of number of genes involved in multiple nutrient stress conditions such as iron, potassium, sodium, and calcium (Hu et al., 2015).

In *Arabidopsis*, the non-protein coding gene IPS1 (Induced by phosphate starvation1) was identified to contain a motif with sequence complementarity to miR399. IPS1 was found to sequester miR399 due to the interrupted pairing of IPS1-miR399. Consequently, overexpression of IPS1 resulted in the reduction of Pi levels in shoots due to the enhanced accumulation of miR399 target PHO2 (Franco-Zorrilla et al., 2007). It is noteworthy to mention that identification and analysis of regulated expression of additional endogenous target mimics will help in developing strategies and approaches to withstand environmental constraints by the plants.

Expression and functional genomics approaches have demonstrated the complex network of regulatory genes involved in Pi-deficiency (Hammond et al., 2003; Wu et al., 2003; Hammond and White, 2008). Under Pi-deficiency, the major transcriptional regulatory system involving PHR1, SIZ1, miR399, and PHO2 has been suggested in *Arabidopsis thaliana* (Fujii et al., 2005; Schachtman and Shin, 2007). In addition, PHR1-miR399-PHO2 signaling pathway has also been shown to operate in rice in response to Pi-deficiency. *OsPHR2*, the homolog of *AtPHR1*, is a key regulator involved in Pi-starvation signaling in rice (Zhou et al., 2008). A recent study suggests that under Pi-deficiency, AtMYB2 regulates miR399f expression by directly binding to MYB-binding site in the promoter region of the miR399f precursor. The over expression of AtMYB2 also affects root system architecture causing reduction in the primary root growth and enhancement in root hair development (Baek et al., 2013).

Interestingly, Pi-responsive miR399 was observed to be induced by *Candidatus liberibacter* infection which causes Huanglongbing (HLB) disease of citrus. sRNA profiling of infected and healthy sweet orange plants identified number of miRNAs and siRNAs induced in response to the infection. The induction of miR399 is in correspondence to Pi-deficiency in the infected plants as compared to the healthy plants (Zhao et al., 2013). This suggests existence of interplay of sRNAs under nutrient deficiency and biotic stresses in plants.

Various studies demonstrated that miR827 and miR2111 are induced in response to phosphate starvation but not under other nutrient deficiency conditions (Hsieh et al., 2009). miR827 target gene encoding proteins containing SPX domain, which is involved in Pi-sensing, and transport in yeast and xylem loading in plants (Hackenberg et al., 2013). miR2111 target gene encodes F-box protein, which is a component of SCF ubiquitin ligase complexes. Notably, all the three inducible miRNAs (miR399, miR827, and miR2111) target genes involved in the ubiquitin-mediated protein degradation pathway, which suggest that the post-translational regulation of genes is a key component in the adaptive response of Pi-deficiency. Intriguingly, phosphate starvation responsive miRNAs such as miR828 regulates ta-siRNAs (*TAS4)* transcript which produces clusters of phased transacting, siRNAs (Hsieh et al., 2009). It has been reported that TAS4-siR81(-), which is one of the dominant *TAS4* siRNAs, targets the transcripts of a group of MYB transcription factors involved in anthocyanin biosynthesis (Rajagopalan et al., 2006). Anthocyanin accumulation is a common stress response and induction of *TAS4-siR81(-)* under N deficiency indicates autoregulation mechanism in plants.

## Sulfur (S)

### Uptake and Transport System

Plants take up sulfur in the form of sulfate (SO_4_^2–^) from the soil via sulfate transporters located on the epidermal and cortical plasma membrane of the roots (Liu et al., 2009). Sulfate transporter gene family has been characterized in different plant species (Takahashi, 2010; Kumar et al., 2011, 2015). *Arabidopsis* genome encodes 14 sulfate transporters, which are divided into five groups on the basis of their sequence similarity, substrate affinity, and tissue specific localization (Grossman and Takahashi, 2001; Kumar et al., 2011). Group 1 transporters are high affinity sulfate transporters involved in the uptake of sulfate from the soil (Howarth et al., 2003; Yoshimoto et al., 2007). Group 2 transporters are low affinity sulfate transporters responsible for the long-distance transport of sulfate (Takahashi, 2010). Group 3 comprises transporters that work in cooperation with low affinity transporters and mainly translocate sulfate from root to shoot (Kataoka et al., 2004). Vacuoles are considered to be the ‘store house’ of sulfate, and during sulfate limiting condition, sulfate is mobilized from vacuoles to the cytoplasm. The sulfate efflux transporters are located on the tonoplast and are classified in Group 4 (Zuber et al., 2010). The transporters belonging to group 5 are also termed as molybdenum transporters due to their involvement in transport of molybdenum inside the plant (Shinmachi et al., 2010). Under sulfur deficiency, predominantly, the expression of high affinity transporters (Group 1) increases which helps in uptake of sulfate from the soil to maintain sulfate homeostasis inside the plant (Kumar et al., 2011).

After sulfate acquisition, S is assimilated into the plastid by the sulfur assimilation pathway (Jez et al., 2016). Sulfate is converted to adenosine 5′-phosphosulfate (APS) by ATP sulphurylase, the first step of this pathway. Further, sulfate is reduced to sulfite by enzyme APS reductase and subsequently to sulfide by sulfite reductase enzyme. This sulfide is incorporated into cysteine, which is a precursor for various sulfur containing compounds such as phytochelatins, metallothioneins, and glutathiones playing important role in stress tolerance (Leustek and Saito, 1999; Dixit et al., 2015a,b,c).

### Regulation of S Homeostasis by Small RNA

**FIGURE 1:**
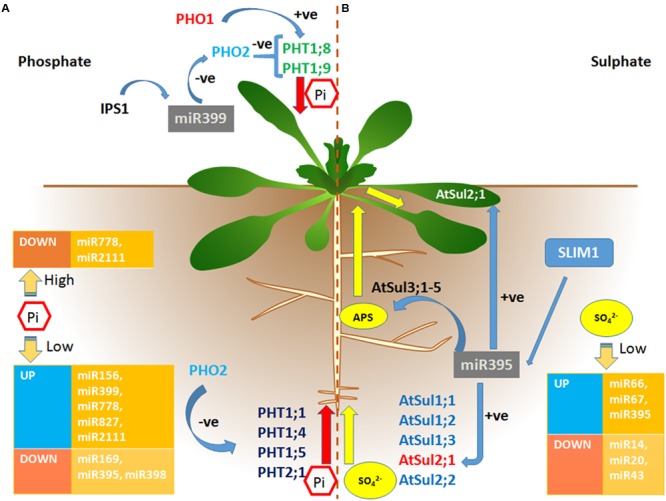
**miRNA mediated regulation of Phosphate and Sulfate uptake, transport and assimilation in plants. (A)** Phosphate (Pi) uptake is regulated by phosphate high affinity transporters. PHO1 and PHO2 are responsible for the xylem loading of Pi. PHO2 act as a negative regulator of Pi loading whereas PHO1 helps in Pi loading into xylem. Various miRNAs regulation under Pi starved and sufficient conditions are shown in orange boxes. The miR399 and isoforms act as primary regulator of Pi uptake, transport and assimilation by negatively regulating PHO2 under Pi starved conditions. Induced by phosphate starvation1 (IPS1) pairs with miR399 and causes its sequestration. **(B)** Sulfate uptake, transport and assimilation involves different transporters. Groups 1 and 2 sulfate transporters are involved in the sulfate uptake from soil to roots. Group 3 is associated with root to shoot transport of sulfate. The expression of various miRNAs under sulfate starved condition has been shown in orange boxes. miR395 has been reported to be sulfate-specific miRNA and acts as a primary regulator of sulfate deficient responsive pathway. Under sulfate deficient condition, miR395 positively regulates expression of a low affinity transporter AtSul2;1 to help in the sulfate uptake and transport to shoot and leaves.

A number of studies have been carried out to identify and validate function of S-responsive miRNAs. These S-responsive miRNAs generally target different transcription factors involved in auxin signaling pathway and stress response (Li et al., 2016) and regulate sulfate uptake, transport and assimilation in plants (**Figure 1**). Deep sequencing identified 27 conserved miRNAs and five novel miRNAs, which express under SO_2_ stress in *Arabidopsis* (Li et al., 2016). The novel miRNAs; miR66 and miR67 were up-regulated more than sixfold whilst miR14, miR20 and miR43 were down-regulated sevenfold in the SO_2_-treated samples in comparison to control (**Table 3**). Comparative deep sequencing of *Arabidopsis* sRNAs treated with different nutrient deficiency including C, N, and S revealed that the targets of differentially expressed miRNAs were related to cellular and metabolic processes, signal transduction, and nutrient homeostasis. miR169b/c, miR826, and miR395 were specifically induced under C, N, and S deficiency, respectively. On the contrary, different miRNAs; miR167, miR172, miR397, miR398, miR399, miR408, miR775, miR827, miR841, miR857, and miR2111 were repressed under the C, N, and S deficient conditions (Liang et al., 2015). Sequencing of sRNA population in *Brassica napus* identified conserved and novel miRNAs responsive to S deficiency and Cd stress (Huang et al., 2010). This indicated that miRNA genes and their corresponding targets coordinately participate in both the stresses. Deep sequencing analysis of miRNAs in *Chlamydomonas reinhardtii* identified differential expression of miRNAs under S deficient and sulfur starved conditions (Shu and Hu, 2012). In addition, studies suggest binding of sulfur-responsive transcription factors particularly, Prohibitin (PHB), Squamosa-Promoter Binding (SPB), and Sulfur Limitation 1 (SLIM1) to the promoter of nutrient responsive miRNAs (Panda and Sunkar, 2015).

**Table 3:** Plant miRNA families responsive to S deficiency.

miRNA families		Plant species	Reference
Up-regulated	Down-regulated		
miR160, miR164, miR169, **miR173**, miR319, miR395, miR400, **miR403**, miR771, miR826, mir829, mir833, miR837, miR846, miR864, mir842, **miR5638, miR8172**	miR167, miR 171, miR172, miR390, miR391, miR397, miR398, miR399, miR408, miR775, miR825, miR827, miR841, miR845, miR850, miR857, miR863, mR1888, miR2111	*Arabidopsis thaliana*	Barciszewska-Pacak et al., 2015; Liang et al., 2015
miR156, miR159, miR164, miR393, miR394, miR395	miR160, miR167, miR168	*Brassica napus*	Huang et al., 2010
miR156, miR159, miR160, miR162, miR164, miR166, miR167, miR168, miR169, miR171, miR172, miR319, miR390, miR393, miR394, miR395, miR396, miR397, miR398, miR399, miR403, miR408, miR530, miR535, miR3627, **miR-c4, miRc-10**		*Carica papaya*	Liang et al., 2013
**miR51, miR62, miR84, miR182, miR196**, miR906, miR909, miR910, miR912, miR914, miR1144, miR1147, miR1148, miR1149, miR1150, miR1153, miR1155, miR1156, miR1158, miR1159, miR1160, miR1164, miR1166, miR1172		*Chlamydomonas reinhardtii*	Shu and Hu, 2012

*Unique miRNAs are represented in **Bold**.*

Intriguingly, miR395 plays an important role in sulfate homeostasis by regulating the expression of genes involved in the sulfate uptake, transport and assimilation (Chiou, 2007; Zeng et al., 2014). It has been observed that miR395 is specifically responsive to S deficiency (Hsieh et al., 2009). The MIR395 loci present in many monocots and dicots express in the vascular system of roots, root tips, and leaves (Kawashima et al., 2009). *Arabidopsis* genome encodes six miR395 genes which are located in two clusters (miR395a,b,c and miR395d,e,f) whereas in rice 24 genes encoding miR395 are clustered into four clusters (Jones-Rhoades and Bartel, 2004; Guddeti et al., 2005).

In *Arabidopsis*, out of four ATP sulphurylases (APSs), APS1,3, and 4 are located in the plastid and APS2 is found in the cytosol (Hatzfeld et al., 2000). miR395 target the mRNAs of three APSs (APS1, APS3, and APS4; **Figure 1**) which catalyze the initial activation step of sulfate assimilation into cysteine (Jones-Rhoades and Bartel, 2004; Sunkar et al., 2012). This clearly suggests that miR395 regulates the plastidial sulfate assimilation as sulfate is reduced and assimilated into cysteine in the plastid (Rotte and Leustek, 2000). Sulphation reaction occurs in the cytosol in which 5′-adenylsulfate is used for the synthesis of glucosinolates (Chiou, 2007). Under S deficiency, the regulation of miR395 remains elusive as induction of miR395 represses the expression of APS1 (Jones-Rhoades and Bartel, 2004) whereas in *Arabidopsis* and *Brassica* roots expression of APS1 and APS3 and total APS activity increases. Moreover, transcript level of APS1 decreases twofold in the shoots of sulfate deficient conditions. Apart from regulating S assimilation, miR395 also regulates S uptake by targeting the gene encoding low affinity sulfate transporter Sul2;1 which is localized in the vascular tissues of roots and leaves (Takahashi et al., 2000; Allen et al., 2005; Kruszka et al., 2012; Zeng et al., 2014).

The miR395 overexpression in *Arabidopsis* suppressed the expression of target genes APS1, APS3, APS4, and Sul2;1 (Liang and Yu, 2010). At the same time, overexpression of OsmiRNA395h in tobacco impaired S homeostasis and affected S distribution among leaves of different ages (Yuan et al., 2016). miR395 overexpressing *Brassica napus* transgenic plants accumulate higher biomass and sulfur content in Cd treated plants as compared to wild type. In addition, transgenic plants accumulated high level of Cd, with less translocation from root to shoot suggesting miR395 is involved in detoxification of Cd in *Brassica napus* (Zhang et al., 2013).

Furthermore, the transcription factor of the ethylene-insensitive like (EIL) family, SLIM1, has been observed to directly or indirectly regulate the expression of miR395 (**Figure 1**) to maintain S homeostasis under deficiency (Kawashima et al., 2011; Matthewman et al., 2012). Under S deficiency, GSH level decreases, which further modulates the expression of genes involved in S metabolism and enhances the expression of miR395. As GSH is an important component of cellular redox signaling, involvement of redox signaling was suggested in the induction of miR395 under S deficiency (Jagadeeswaran et al., 2014). In addition, analysis of miRNA395 over expressing *Arabidopsis* plants, *slim1-1* mutants, and plants with reduced miR395 activity by target mimicry depicted the interplay of SLIM1 and miR395 in the sulfate assimilation in *Arabidopsis* (Todesco et al., 2010; Kawashima et al., 2011). Thus, the S-deficiency induced expression of miR395 represents a link between redox signaling and SLIM1 transcription factor.

## Conclusion and Future Perspectives

**FIGURE 2:**
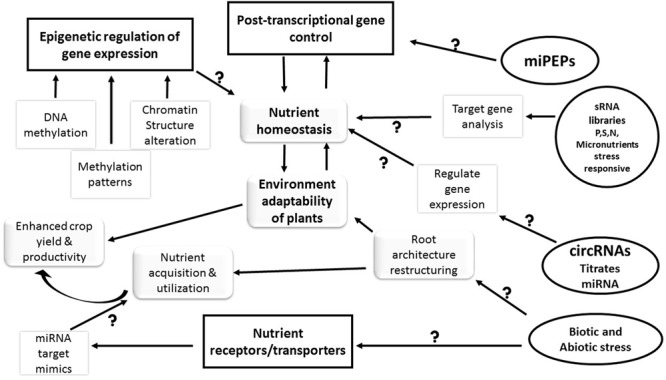
**Future perspectives in regulation of nutrient uptake/transport and assimilation in plants.** The figure illustrates the role of various factors in maintenance of nutrient uptake, assimilation and transport. The post-transcriptional and epigenetic modifications have driven the nutrient sensing, uptake and transport in the plants. However, new perspectives in the nutrient regulation such as miRNA-PEPs (miPEPs), circular RNAs (circRNAs), and micro RNA (miRNA) target mimicry are required to generate more information about the pathways and regulators of nutrient homeostasis in plants. The information acquired in these areas will eventually lead to better nutrient acquisition and utilization by the crop plants. The square boxes are the known factors whereas the factors in circular/oval boxes represent the knowledge voids (questions marks) that need further investigation in near future. The arrows represent the relationship between the interconnected events.

Plants as sessile organisms have evolved several mechanisms to fulfill any type of nutrient deficiency. The roots and other aerial parts of the plant act as an extension for various signaling cascades to form a nexus to adapt to nutrient stress. miRNAs being an important component of this nexus, have been found to be riboregulatory in regulation of nutrient sensing, transport and assimilation, such as miR395 and miR399 for S and P, respectively. In plants, though lesser number of miRNA gene clusters exist, existence of miR399 and miR395 clusters reflect the occurrence of gene duplication events during evolution. This may be the reason for the coordinated regulation of these miRNAs under nutrient deficiency in plants. Though, a large number of nutrient deficiency responsive miRNAs have been identified, role of these miRNAs in regulating the nutrient stress needs to be studied. To elucidate various components and networks involved in nutrient homeostasis in plants, there is need to study different regulatory aspects (**Figure 2**) in detail. Future studies required in these areas are summarized below.

Apart from genetic regulation of gene expression, the epigenetic regulation also occurs in plants to counter nutrient deficiency as well as sufficiency. Studies have identified that alteration in chromatin structure and methylation pattern govern environmental adaptability in plants under nutrient deficiency (Sirohi et al., 2016). It has been reported that miRNAs also regulate gene expression via DNA methylation. For example, miR165/166 regulate the expression of target genes by DNA methylation (Bao et al., 2004). Thus, investigation of epigenetic control during nutrient deficiency can offer clear and insightful information about the plant adaptability toward environmental constraints.

The recent study in the field of plant interactome suggested an insight into the global organization of various biological processes that constitute a community network of different hypothetical functional links between proteins and pathways (Arabidopsis Interactome Mapping Consortium, 2011; Reichel et al., 2016). Such studies are required to establish sRNA-protein and protein-protein interaction networks to understand sRNA-mediated regulation and dynamic rewiring of processes such as nutrient sensing, uptake, transport, assimilation, and interactions occurring in plants.

In-depth sequencing of sRNA libraries and target gene analysis under single or multiple nutrient stress might help in the better understanding the cohort of sRNA-mediated stress responses in plant. Identification and characterization of endogenous target mimics for nutrient responsive miRNAs may provide deeper understanding about nutrient acquisition and utilization in plants. Recently, miRNA-PEPs (miPEP) have been shown to regulate a number of miRNAs (Lauressergues et al., 2015; Couzigou et al., 2016). It will be interesting to study whether such miPEPs are encoded by MIR genes responsive to nutrient deficiency and play role in nutrient homeostasis. The circular RNAs (circRNAs), a product of back-splicing of precursor mRNA; interfering eukaryotic processes by interference of splicing and transcription and also titrates miRNAs has been reported to regulate and reshape the gene expression (Lu et al., 2015; Wang and Wang, 2015; Chen, 2016). Thus, a combination of various different approaches using high throughput technologies could help us uncover the master regulators and deregulators of plant nutrient stress.

An insight into the complexity of nutrient-plant interaction, the root system restructuring and the nutrient stress is essential for the improvement of crop yield and productivity as these depend upon ability of plant to utilize surrounding nutrients. The *in situ* imaging studies employing non-destructive X-ray based techniques (Perret et al., 2007) will be useful in elucidation of the root system growth dynamics and their restructuring upon various nutrient stresses. This will provide a que for plant breeders in future to develop hybrids with well-developed root architecture that might withstand adversity against many nutrient stresses and drought conditions. Thus, the exploration of precise mechanisms involved in sensing, signaling, and cross-talk of nutrients and miRNAs will help in developing strategies for improving the nutrient use efficiency and increasing crop productivity required for the global sustainability and food security.

## Author Contributions

SK and PT conceived the idea and planned the manuscript. SK, SV, and PT wrote the manuscript.

## Conflict of Interest Statement

The authors declare that the research was conducted in the absence of any commercial or financial relationships that could be construed as a potential conflict of interest.
